# The role of virtual sub-internships in influencing career perceptions: an international medical graduate perspective

**DOI:** 10.1080/10872981.2020.1821463

**Published:** 2020-09-14

**Authors:** Rucira Ooi, Setthasorn Zhi Yang Ooi

**Affiliations:** a Department of Obstetrics and Gynaecology, Princess of Wales Hospital, Bridgend, UK; b Cardiff University School of Medicine, University Hospital of Wales, Cardiff, UK

**Keywords:** International medical graduate, sub-internship, career perception, residency, application

## Abstract

The SARS-CoV-2 pandemic has left a huge impact on the career perceptions of trainees and medical students globally. The cancellation and/or replacement of sub-internships in the United States (US) with a virtual alternative offer a different experience. We explore the impact of this issue on international medical graduates (IMGs) who are planning to apply to a US residency program in the near future.

Dear Editor,

The article by Byrnes et al. has inspired us to consider the effect of the SARS-CoV-2 virus pandemic on career perceptions of international medical graduates (IMGs). Byrnes et al. demonstrated that the career perceptions of United States (US) medical students are not affected by the loss of sub-internship [1]. Although the article reflects the perspective of medical students in the US, we would also like to share the impact of canceled sub-internships on career perceptions for IMGs.

Sub-internships are one of the main highlights of training in medical school. For IMGs planning to apply to a residency program in the US, sub-internships in the US have been considered the stepping stone to a successful residency program application. This is an opportunity for IMGs in their final year of medical school to learn about specific residency programs in the US and assess whether they are a ‘good fit’ for the program before applying for residency in the future. The cancelation of sub-internships would mean that IMGs will not have the opportunity to build meaningful relationships with the doctors in the program, demonstrate their level of competency in clinical knowledge and interpersonal skills, nor gain mentorship from doctors who will provide letters of recommendation at the time of application. Letters of recommendation are especially important in reflecting a mentor’s perception of an applicant’s performance as a clinician and an academic. This serves as a key source of information about the distinguishing personal qualities of applicants [2]. While the curriculum vitae (CV) and personal statements have many similarities from candidate to candidate, letters of recommendation are an opportunity to qualify the factors that set applicants apart [2]. These factors could be a setback to IMGs who plan to pursue a residency in the US, hence causing them to change their career paths.

Fortunately, some programs have organized virtual sub-internships as a replacement for the loss of the in-person experience. Virtual sub-internships are a series of webinars organized by current residents and program directors to give the audience insight into the nature, ethos, and values of the program, hospital, and team members.

While virtual sub-internships are the next best alternative to in-person sub-internship, this letter aims to explore the pros and cons of virtual sub-internships that may affect the career perceptions of IMGs.

## Pros

1. Students can attend many virtual sub-internships and learn in-depth about the residency programs offered at multiple different hospitals within a similar time frame to an in-person sub-internship. As a consequence, students will be able to make a more informed choice to which residency program they plan to pursue in the future.
2. IMGs will still be able to gain the contacts of the doctors, hence, allowing them the opportunity to correspond through email. IMGs can take this opportunity to build their network with the doctors, which will potentially allow them to arrange future pre-residency fellowships or pursue academic work alongside these new connections.
3. There are no pre-requirements to attend a virtual sub-internship. Unlike in-person sub-internships, IMGs are not limited to the programs that are only affiliated with their medical schools. Moreover, non-final year medical students are also able to participate virtually in anticipation of applying for an in-person sub-internship in the future.
4. Virtual sub-internships are free of charge and can be accessed at the comfort of the student’s own home.

## Cons

1. The value of virtual sub-internship cannot match the standard of an in-person sub-internship. While there are many advantages of a virtual sub-internship, it does not provide participants with the necessary clinical exposure. Medical students, too, are unable to demonstrate their level of competency in clinical or interpersonal skills, nor will they have the opportunity to work towards a letter of recommendation.
2. The process of building connections and gaining personal mentorship will take longer as compared to an in-person sub-internship.
3. IMGs may attempt to send ‘cold emails’ to the doctors in the program, but the success rate of ‘cold emails’ is low.
4. The experience from a virtual sub-internship is one-sided and is not personal.

Despite the added challenges on career perceptions experienced by IMGs during this pandemic, not all is lost. Everyone is doing their best to accommodate the ‘new normal’. The effects of the Covid-19 pandemic on hospitals and frontline workers cannot be ignored, and the safety of all staff and patients is the priority.

We hope that the residency program admission committees will be more understanding of the circumstances of IMGs during this pandemic. On the other hand, IMGs should still attempt to partake in any local clinical experiences available. Through this, IMGs can build their connections locally to gain the support they need to secure a pre-residency fellowship, which will be beneficial towards their residency application.

Unprecedented times such as the current pandemic may challenge one’s career perceptions. However, IMGs should remain hopeful and continue to pursue their career aspirations.
